# Providing the right care to patients admitted in Intensive care units in low-and middle-income countries: The case of Nepal

**DOI:** 10.7189/jogh.13.03032

**Published:** 2023-06-30

**Authors:** Dejina Thapa, Hon Lon Tam, Sek Ying Chair, Subhash Prasad Acharya

**Affiliations:** 1 The Nethersole School of Nursing, Faculty of Medicine, The Chinese University of Hong Kong, Shatin, N.T., Hong Kong SAR, The People’s Republic of China; 2 Department of Critical Care Medicine, Maharajgunj Medical Campus, Institute of Medicine, Tribhuvan University, Kathmandu, Nepal

**Figure:**
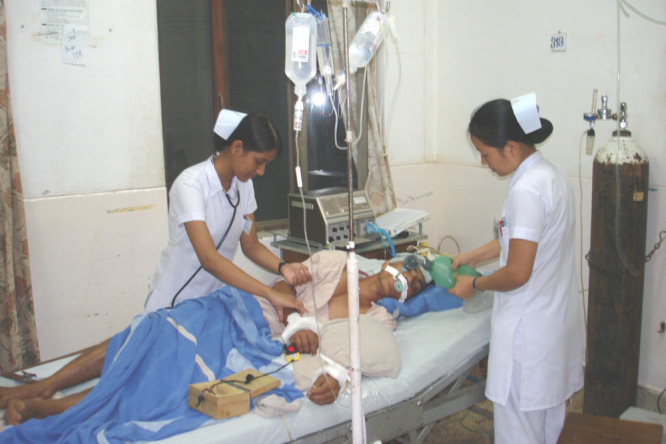
Photo: Critically ill patients are being treated in the general ward in rural parts of Nepal where there is a lack of intensive care unit facilities. Source: The Hospital at the End of the World”, used with permission.

“The patient’s condition is very critical, hurry up!!! The patient needs a mechanical ventilator; we must transfer the patient to the intensive care unit immediately”; a frequently heard chaos in hospitals all over the world. Such patients are “critically ill” which is the life-threatening stage of acute illness that requires prompt attention. Thus, providing the right critical care at the right time at the right place are essential for reducing mortality and morbidity. The right place for providing the right care to the mechanical ventilated patient is the “Intensive care unit” (ICU). World Health Organization (WHO) commission has highlighted the urgent need for all nations to strengthen health systems’ capacity on delivering appropriate, affordable care around the globe [1]. Providing acute care to critically ill patients is a global enterprise, regardless of health system capacity. However, 8.6 million people die prematurely in low- and middle-income countries (LMICs) because of an inefficient health care system and delayed treatment. Improving the quality and accessibility of critical illness care in LMICs is crucial for reducing this burden [2]. Despite the fact, critical care in Nepal is one of the most critically under-prioritized areas in the health system.

Several emerging trends exits in health care, including an increase in demand for critical care services. For the aging population, Global Health and Aging research estimates that the number of people 65 and older will increase by 1.5 billion in 2050, with a majority of growth in LMICs [3]. Furthermore, the prevalence of disease and co-morbid disorder has increased; accounting for 66% of all fatalities in Nepal, according to the Nepal Burden of Disease 2017 report, with injuries accounting for an additional 9% [4]. Theses co-morbid diseases such as hypertension, diabetes, and cardiovascular disease are the major reasons for ICU admission in Nepal. This trend will increase the demand for acute curative services for life-threatening situations, and requiring immediate attention.

Nepal is a low-income country, with roughly one-quarter of the population living below the poverty line and total health care spending as low as 6% of gross domestic product. ICU stays in Nepal typically cost between 100 US dollars (US$) and US$200 per day [5]. Private ICUs are more expensive compared to public ICUs, costing three to five times more. In addition, critical care costs are often not fully covered by health insurance, resulting in significant out-of-pocket expenses for patients and their families [5]. This makes it difficult for Nepalese individuals with low socioeconomic status to access ICU care due to the high costs. It is common for people to take extreme measures, such as selling their land, taking out loans, discontinue medical care, and even committing suicide, to pay for their health care expenses [5]. Each year, high health care costs could push an additional 800 000 people into poverty [6].

Although the critical care specialty in Nepal has grown significantly over the last decade, after the establishment of the Nepalese Society of Critical Care Medicine, Nepal Critical Care Development Foundation, and the Critical Care Nursing Association of Nepal. Yet, there are still many challenges to delivering high-quality critical care without financial burden to the entire population. According to Nepal's Government 2015 guidelines, hospitals with 50 or more beds should have at least 5% ICU beds. One ventilator per two ICU beds, a 1:1 nurse-to-patient ratio, and isolation beds for infectious disease patients are also needed. However, not every hospital in the nation now has an ICU facility as per the aforementioned recommendation. A recent study by Neupane and colleagues reported approximately 6% of all hospital beds are ICU beds, among them 3.2% of the beds are ventilator-equipped ICU beds., meaning that only 2.5/100 000 patients could be admitted in an ICU bed [7]. Therefore, ICU beds in public hospitals are often occupied, which results in delays in transfer to the ICU, which is associated with an increase in the untimely dismiss of a patient [5]. It speaks of rising incidents of increasing violence against doctors and vandalism in hospitals by their relatives with some ending in physical assault. Furthermore, there is a shortage of trained doctors and nurses with specialized training. Because, the global attraction of monetary gains causes brain drain to developed countries, thus there is a constant shortage of human resources [5]. In addition, multidisciplinary care, in which a patient is treated by nutritionists, physiotherapists, clinical pharmacists, and social workers, is only practiced in large, tertiary care centres in private and academic medical institutions [7]. Next, there is a lack of local epidemiological data, including ICU capacity, life-saving medical resources, cost-effective critical care, ICU cases, the process of care, staffing pattern, and mortality [7]. Since the establishment of the Nepal Intensive Care Research Foundation in 2020, research has continued. Unfortunately, only twelve hospitals participated in this study. To obtain a nationwide survey, there is still much work to be performed.

Unfortunately, critical care in rural Nepal is not well developed. It is still in the incipient stage where a large portion of the Nepalese population lives. The rural hospitals do not have critical care units and seriously ill patients are treated in the general wards or often referred to other wards. Many districts of Nepal, especially in the mid-west and far west, are lack of road links to the district hospital, and where roads exist, they are rough or impassable. Transferring critically ill patients to well-equipped critical care hospitals is challenging in these districts because they lack even a single ambulance. In addition, basic medical equipment are also scarce in rural hospitals, such as mechanical ventilators for patients with respiratory failure, emergency lifesaving drugs and antibiotics, and diagnostic equipment like arterial blood gas machines [7]. Moreover, maintaining expensive ICU equipment in remote locations, where there may be frequent power outages and a lack of experienced maintenance people, where equipment frequently breaks down or cannot be used to its full potential, is one of the biggest obstacles. Furthermore, inadequate infection control measures and improperly trained workers may frequently lead to more significant problems, which could worsen outcomes and raise costs even more. Because of this, people are losing their lives the vulnerable age groups, especially children, and the underprivileged are the main victims [8].

Nepal must overcome significant obstacles to improve critical care throughout the country. Understanding the true burden of critical illness in resource-limited settings is challenging, hindering both local and global appreciation. However, constructing new ICUs immediately is a challenging task for a country like Nepal. A wise place to start would be to integrate care with existing services. Well-managed triage protocols in the emergency department can help screen patients for ICU admission; converting postoperative beds into ICU beds is a workable option. An educational initiative could be an additional solution. Short-term courses to longer-term initiatives are based on academic partnerships between high-income and low-income countries (including academic institutions, professional associations, and international non-government organisations). The Rwanda Human Resources for Health Program is a very good initiation of academic collaboration: A collaboration between 25 American organizations and the Rwandan Ministry of Health where it trained 500 specialist- physicians [9]. These academic collaborations can serve as platforms for knowledge exchange and the development of local ICU faculty expertise. The hiring of health care professionals and other human resources must also be planned for and financed following a long-term vision. Additionally, from a research standpoint, each ICU should use the data registry of all the epidemiological characteristics and ICU outcomes. The establishment of mutual coordination between the health ministry and its divisions, government, private, and non-governmental organizations should be prioritized above all else. Managers of health care organizations must be accountable and adopt efficient behaviours to build trust with global health donors and obtain adequate funding [10]. Organizations can be persuaded to strengthen health systems by fostering an environment that encourages local engagement and learning. Another good example of strong managerial leadership from countries like Ethiopia and Kyrgyzstan, is that these countries have developed the national ownership for development plans with support from external donors, and increased donor coordination [9,11]. This approach would align with the WHO “health system strengthening” for the development of the strategies and roadmaps towards universal health coverage. These strategies should be accompanied to improve the critical care services in Nepal.

Thus, critical care in Nepal is developing. There has been progress, but there is still much to be done. The field is bursting with opportunities, challenges, and dynamism. Ultimately, it is hoped that all of this work will provide a service that is both scientific and meaningful to the many critically ill patients.
